# The digital transformation in pharmacy: embracing online platforms and the cosmeceutical paradigm shift

**DOI:** 10.1186/s41043-024-00550-2

**Published:** 2024-05-08

**Authors:** Ahmad Almeman

**Affiliations:** Department of Pharmacology, College of Medicine, Qassim University, Buraydah, Saudi Arabia

**Keywords:** Digital transformation, Healthcare technology, Cosmeceuticals, Global health, Patient care, Sars-cov-2, 2019 novel coronavirus, Drug safety

## Abstract

In the face of rapid technological advancement, the pharmacy sector is undergoing a significant digital transformation. This review explores the transformative impact of digitalization in the global pharmacy sector. We illustrated how advancements in technologies like artificial intelligence, blockchain, and online platforms are reshaping pharmacy services and education. The paper provides a comprehensive overview of the growth of online pharmacy platforms and the pivotal role of telepharmacy and telehealth during the COVID-19 pandemic. Additionally, it discusses the burgeoning cosmeceutical market within online pharmacies, the regulatory challenges faced globally, and the private sector’s influence on healthcare technology. Conclusively, the paper highlights future trends and technological innovations, underscoring the dynamic evolution of the pharmacy landscape in response to digital transformation.

## Introduction

Digital technology is driving a massive shift in the worldwide pharmacy industry with the goal of improving productivity, efficiency, and flexibility in healthcare delivery. In the pharmacy industry, implementing digital technologies like automation, computerization, and robotics is essential to cutting expenses and enhancing service delivery​​ [1]. With a predicted 14.42% annual growth rate, the digital pharmacy market is expanding significantly and is expected to reach a market volume of about $35.33 billion by 2026. This expansion reflects the pharmacy industry’s growing reliance on and promise for digital technologies​ [2].

Pharmacy services have always been focused on face-to-face communication and paper-based procedures. However, the drive for more effective, transparent, and patient-centered healthcare is clear evidence of the growing need for digital transformation. Breakthroughs like mobile communications, cloud computing, advanced analytics, and the Internet of Things (IoT) are reshaping the healthcare sector. These breakthroughs have the potential to greatly improve patient care and service delivery, as demonstrated in other industries including banking, retail, and media [3].

In the pharmacy industry, a number of significant factors are hastening this digital transition. Important concerns include the desire for cost-effectiveness, enhanced patient care, and more transparency and efficiency in medication development and manufacture. This change has been made even more rapid by the COVID-19 pandemic, which has highlighted the necessity for digital solutions to address the difficulties associated with providing healthcare in emergency situations [4].

In terms of specific technologies being adopted, artificial intelligence (AI) and machine learning are playing a pivotal role. The McKinsey Global Institute estimates that AI in the pharmaceutical industry could generate nearly $100 billion annually across the U.S. healthcare system. The use of AI and machine learning enhances decision-making, optimizes innovation, and improves the efficiency of research and clinical trials. This results in more effective patient care and a more streamlined drug development process​ [5].

The digital transformation in the pharmacy sector represents a pivotal shift in the delivery and experience of healthcare services. This evolution is more than a transient trend; it’s a fundamental alteration in the healthcare landscape [6]. The adoption of digital technologies is reshaping aspects of healthcare, including patient engagement and medication adherence, leading to enhanced healthcare outcomes. Research indicates that digital tools in pharmacy practices have resulted in more individualized and efficient patient care. Telehealth platforms, exemplified by companies like HealthTap, are being increasingly incorporated by pharmacies to augment patient care via technological solutions. The contribution of digital health technology to medication adherence is notable, employing a variety of tools such as SMS, mobile applications, and innovative devices like virtual pillboxes and intelligent pill bottles. These advancements are pivotal in addressing the critical issue of medication nonadherence in healthcare. Furthermore, digital health tools are empowering pharmacists with expanded clinical responsibilities, particularly in the management of chronic diseases like diabetes, where apps and smart devices provide essential features such as blood glucose tracking and medication reminders. This comprehensive integration of digital health into pharmacy practice signifies a transformative era in healthcare delivery and patient management [7].

**Fig. 1:**
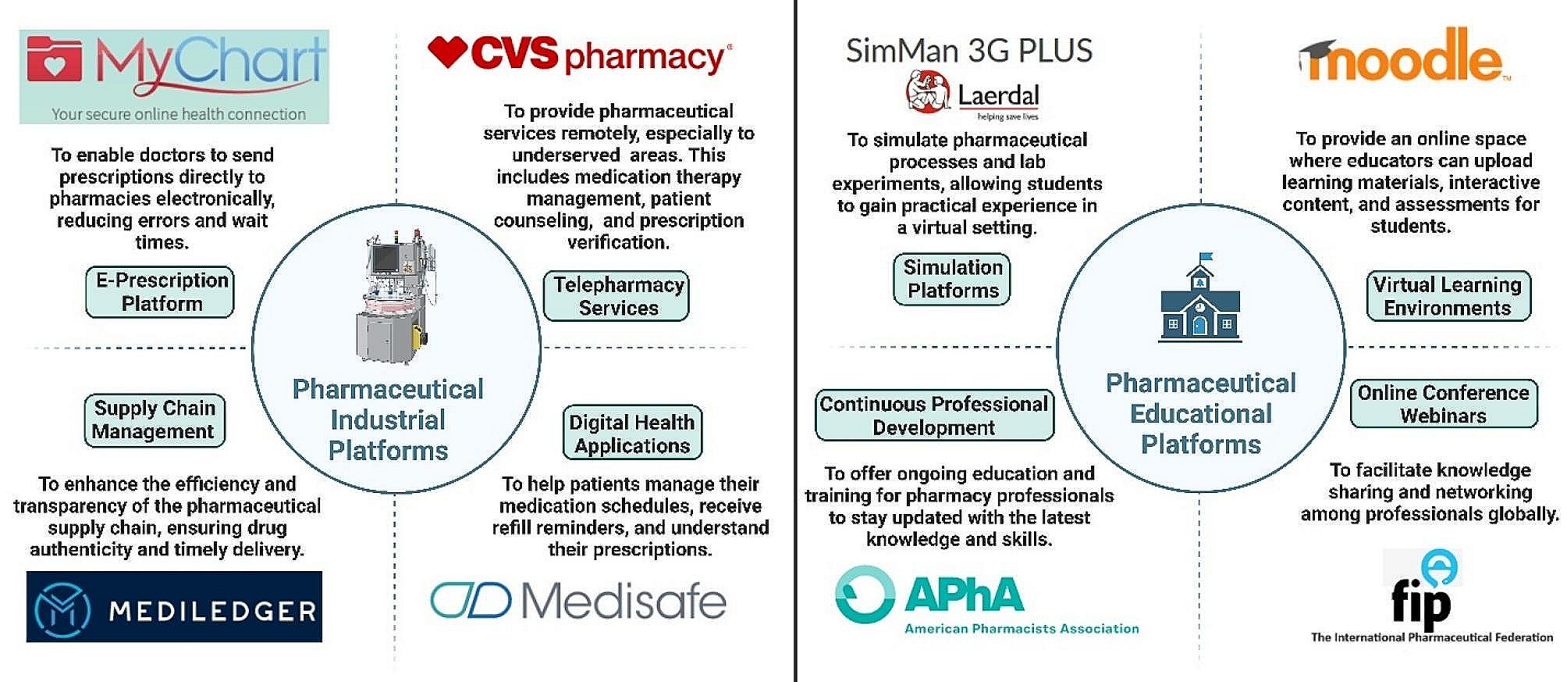
Comprehensive overview of different platforms in the pharmaceutical industry and education illustrating purposes and exemplary cases

Online platforms are being used increasingly by the pharmaceutical sector and educational institutions to improve efficiency, flexibility, and accessibility. The telepharmacy program at CVS Pharmacy is an example of how telepharmacy services, which provide remote counseling and prescription verification, bring pharmaceutical care to underprivileged communities [8]. Prescription accuracy and drug management are enhanced by e-prescribing software like Epic’s MyChart and digital health apps like Medisafe [9; 10]. Blockchain technology is also being investigated for transparent and safe supply chain management. Continuous learning and professional networking are made possible in education by Virtual Learning Environments (VLEs) like Moodle [11], simulation software like SimMan 3G Plus [12], Continuing Professional Development (CPD) platforms like the American Pharmacists Association [13], and online conference platforms, as shown in Fig. 1. While these platforms offer significant benefits like enhanced access and cost-effectiveness, they also present challenges, including addressing the digital divide and ensuring the quality and credibility of online services to maintain professional standards and patient safety.

In this review, we summarized the digital transformation in the pharmacy sector, emphasizing the integration of online platforms and the emerging significance of cosmeceuticals. We discussed the global shift towards digital healthcare, including telehealth and online pharmacy services, and how these changes have been accelerated by the COVID-19 pandemic. The paper also examined the impact of digital technologies on pharmacy practice and education, with a focus on telepharmacy services, e-prescribing software, and digital health apps. Additionally, it addresses the challenges and opportunities presented by this transformation, including regulatory and safety concerns, and the need for continuous professional development in the digital era.

## The global impact of online pharmacy platforms

In recent years, the landscape of pharmacy practice and education has undergone a significant transformation, driven by technological advancements and catalyzed by the global COVID-19 pandemic. A study highlighting the increasing consumer trust in online medication purchases pre, during, and post-pandemic reveals a shift in consumer behavior towards online pharmacies [14]. This trend underscores a greater reliance on these platforms, where the perceived benefits significantly outweigh the perceived risks, indicating a positive reception and growing trust in digital healthcare solutions.

The adoption of telehealth, including telepharmacy, exemplifies this shift. In the United States, patient adoption of telehealth services surged from 11% in 2019 to 46%, with healthcare providers expanding their telehealth visits [15]. This shift is a reflection of how adaptable the healthcare sector is to digital platforms and how customer acceptance is increasing. The epidemic has also served as a catalyst, hastening the creation and uptake of online telepharmacy services throughout the world. The “new normal” has forced the addition of new platforms to support established sources of health information. The creation and evaluation of an online telepharmacy service in the Philippines during the pandemic serves as an example of this, demonstrating how quickly the global pharmacy industry adopted digital solutions. These services are essential for providing and elucidating pharmaceutical information within the context of primary healthcare delivery; they are not merely supplementary [16].

Simultaneously, pharmacist-led companies such as MedEssist and MedMehave, innovated digital platforms to facilitate services like flu shots or COVID-19 tests, reflecting a move towards customer-centric, digital-first services [17]. This transition enhances convenience and access to care but also introduces significant regulatory challenges. As the growth of online medicine sales disrupts traditional pharmacy markets, navigating these challenges becomes crucial for maintaining patient safety, quality standards, and fostering a trustworthy online healthcare environment [18].

Parallel to the practice changes, educational platforms for pharmacy have also evolved, especially under the impetus of the pandemic. These platforms have integrated a mix of traditional and student-centered teaching methodologies, including remote didactic lectures and on-site experiential training. The implementation of blended learning approaches, which combine remote lectures with on-site laboratory classes, reflects a broader educational trend towards hybrid models. This approach aims to leverage the advantages of both online and traditional methods, offering a more flexible and potentially more effective educational experience [19].

It takes more than just implementing new tools to integrate educational technology into pharmacy education, it also requires understanding how these technologies affect instruction and student learning. To effectively improve the educational experience, their utilization must have a purpose. There is an increasing amount of scholarly interest in this field, as evidenced by systematic reviews of the effects of new technologies on undergraduate pharmacy teaching and learning [20]. These digital platforms will probably become more significant in the future of pharmacy education, helping to mold the profession and guaranteeing that pharmacists are equipped to fulfill the ever-changing demands of the healthcare system. This development is indicative of a larger trend in the healthcare industry toward a more flexible, patient-focused, and technologically advanced environment [21].

## Digital transformation in global healthcare

The recent advancements in digital transformation within global healthcare are significantly reshaping the landscape of healthcare and pharmacy services. These transformations are largely driven by the integration of digital technologies, which are redefining the tools and methods used in health, medicine, and biomedical science, ultimately aiming to create a healthier future for people worldwide [22]. In a 2018 report [23], Amazon’s potential entry into the $500 billion U.S. pharmacy market, the second-largest retail category, through mail-order and online pharmacies was highlighted as a significant industry disruptor. With licenses in at least 12 states in the US and a strategy focused on bypassing middlemen, Amazon’s historical success positions it to transform the pharmacy landscape, promising enhanced efficiency and cost savings for consumers.

One of the critical areas identified in recent research is the establishment of five priorities of e-health policy making: strategy, consensus-building, decision-making, implementation, and evaluation. These priorities emerged from stakeholders’ perceptions and are crucial for the effective integration and adoption of digital health technologies​ [24]. This holistic approach is increasingly relevant for scholars and practitioners, suggesting a focus on how multiple stakeholders implement digital technologies for management and business purposes in the healthcare sector [25]​​. The deployment of technological modalities, encompassed within five distinct clusters, can facilitate the development of a digital transformation model. This model ensures operational efficiency through several dimensions: enhanced operational efficacy by healthcare providers, the adoption of patient-centered methodologies, the integration of organizational factors and managerial implications, the refinement of workforce practices, and the consideration of socio-economic factors [25].

Studies focusing on value creation through digital means suggest healthcare as a consumer-centric realm ripe for center-edge transformations, characterized by self-service and feedback cycles. These transformations are vital in addressing inherent tensions between patients and physicians, steering the focus towards value co-creation and service-dominant logic [26]. Participatory design and decision-making approaches are emphasized for enhancing health information technology’s performance and institutional healthcare innovation. Such approaches are particularly crucial in developing national electronic medical record systems and improving chronic disease treatment through electronic health records. Additionally, telehealth research integrates patients’ perceptions, contributing to the understanding of technology, bureaucracy, and professionalism within healthcare [27].

The impact of health information technology (HIT) on operational efficiencies is profound. Empirical studies, such as those by Hong and Lee [28], Laurenza et al. [29], and Mazor et al. [30], demonstrate positive correlations between HIT and patient satisfaction, quality of care, and operational efficiency. However, challenges remain, as Rubbio et al. [31] highlight deficiencies in resilience-oriented practices for patient safety. Organizational and managerial factors in digital healthcare transformation also receive significant attention. Hikmet et al. [32] and Agarwal et al. [33] investigate the role of organizational variables and barriers in HIT adoption, whereas Cucciniello et al. [34] delve into the interdependence between implementing electronic medical records (EMR) systems and organizational conditions. Further, Eden et al. [35] and Huber and Gärtner [36] explore workforce adaptations and the implications of health information systems in hospitals that can increases transparency of work processes and accountability. Lastly, examining healthcare financialization and digital division provides an international perspective, contrasting the regulated environment in the EU with the US’s use of online medical crowdfunding as a potential solution to reduce bankruptcy [37; 38]. Collectively, these studies suggest a comprehensive model where stakeholders leverage digital transformation for management, enhancing operational efficiency in healthcare service providers.

**Fig. 2:**
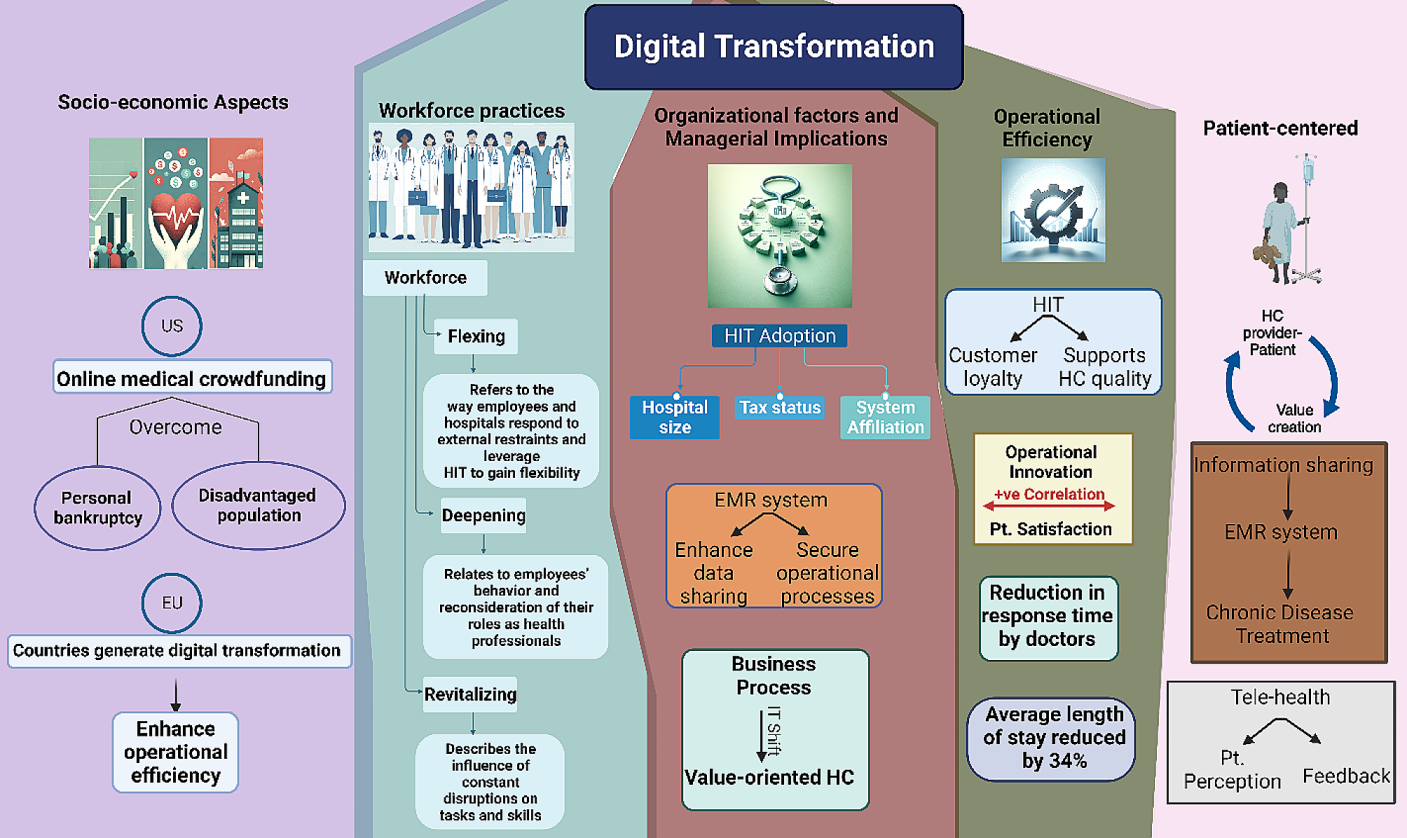
Visual representation of multifaceted digital transformation in healthcare: a synthesis of provider-patient dynamics, HIT impact, and strategic management. HIT; health information technology, HC; healthcare, EMR; electronic medical records. IT; information technology, Pt.; patient

Marques and Ferreira [39] performed a systematic literature review of digital transformation in healthcare, spanning the period from 1973 to 2018. Utilizing the SMARTER (Simple Multi-attribute Rating Technique Exploiting Ranks) method, 749 potential articles were analyzed, culminating in the prioritization and selection of 53 articles for detailed examination. The literature was organized into seven thematic areas: (1) Integrated management of IT in healthcare, (2) Medical images, (3) Electronic medical records, (4) IT and portable devices in healthcare, (5) Access to e-health, (6) Telemedicine, and (7) Privacy of medical data. It was observed that the predominant focus of research resides in the domains of integrated management, electronic medical records, and medical images. Concurrently, emerging trends were identified, notably the utilization of portable devices, the proliferation of virtual services, and the escalating concerns surrounding privacy. See Fig. 2 for visual representation of multifaceted digital transformation in healthcare.

## Telehealth and online pharmacy advancements in pandemic management

In the realm of online pharmacies and telehealth, digital health technologies have been instrumental in managing the COVID-19 pandemic through surveillance, contact tracing, diagnosis, treatment, and prevention. These technologies ensure that healthcare, including pharmacy services, is delivered more effectively, addressing the challenges of accessibility and timely care. The role of telemedicine and e-pharmacies, in particular, has been emphasized in improving access to care worldwide. By enabling remote consultations and drug delivery, these platforms are making healthcare more accessible, especially in regions where traditional healthcare infrastructure is limited or overstretched [40].

The Canadian Virtual Care Policy Framework advocates for the swift adoption and integration of virtual care, propelled by the COVID-19 pandemic. It emphasizes enhancing access and quality, ensuring equity and privacy, and devising appropriate remuneration models, employing a collaborative, patient-centered approach while addressing digital disparities. During the COVID-19 pandemic, Canadian provinces and territories rapidly adopted virtual health care, leading to 60% of visits being virtual by April 2020, up from 10 to 20% in 2019. However, these implementations were often temporary and not fully integrated into healthcare systems. By August 2020, virtual visits decreased to 40%, with variations across regions, while provinces and territories used temporary billing codes for these services. The framework’s “Diagnostique” provides a thorough analysis of policy enablers and strategies for virtual care, underscoring the need for comprehensive policy and partnership engagement [41]. In the context of digital transformation in pharmacy, the Hospital News article outlines the application and infrastructure of telepharmacy services in Canada, highlighting the geographical challenges and the early adoption of telepharmacy in certain regions since 2003. It notes the use of various technologies like Medication Order Management, Videoconferencing, and Remote Camera Verification. Although lacking specific quantitative data, the article underscores the necessity for expanded telepharmacy services to ensure uniform care quality across diverse locations [42].

Similarly, Telehealth offers extensive resources for patients and providers in the United States, emphasizing programs like the Affordable Connectivity Program and Lifeline to facilitate access. The Health Resources and Services Administration enhances telehealth through support services, research, and technical assistance, reflecting a significant outreach impact [43]. The Office for the Advancement of Telehealth (OAT) under Health Resources and Services Administration (HRSA) works to improve access to quality health care through integrated telehealth services in the US. It supports direct services, research, and technical assistance, with over 6,000 telehealth technical assistance requests sent to Telehealth Resource Centers and approximately 22,000 patients served [44].

Internationally, In the UK, the National Health Service (NHS) spearheads digital health and care, providing significant innovation opportunities through vast data management. Support for digital health spans various stages, from discovery with organizations like Biotechnology and Biological Sciences Research Council (BBSRC) and Intelligent Data Analysis (IDA) research group, to development with networks such as Catapults and CPRD, and delivery with entities like the Academic Health Science Networks (AHSNs) and DigitalHealth.London. Regulatory bodies like the Medicines and Healthcare products Regulatory Agency (MHRA) and NICE ensure safety and efficacy. The collaborative ecosystem involves academic, healthcare, and industry stakeholders, aiming to enhance health and care services through technology and innovation [45].

In Australia, the government’s investment of over $4 billion into COVID-19 telehealth measures has facilitated universal access to quality healthcare. This initiative has provided over 85 million telehealth services to more than 16 million patients, with approximately 89,000 healthcare providers engaging in this service delivery. From 1 January 2022, telehealth services, initially introduced in response to COVID-19, will become an ongoing part of Medicare. This will allow eligible patients across Australia continued access to general practice (GP), nursing, midwifery, and allied health services via telehealth, deemed clinically appropriate by the health professional [46].

European nations such as the Netherlands, Austria, and Italy are at the forefront of implementing cross-organizational patient records, significantly enhancing telehealth communication and facilitating cross-border healthcare. The role of strong government support in advancing telehealth is pivotal. Ursula von der Leyen, the President of the European Commission, has been a prominent advocate for eHealth. She proposed the establishment of a European Health Data Space to streamline health data exchange across member states. France, a leader in telehealth legislation for nearly a decade, has pioneered a public funding scheme for tele-expertise at a national scale. Despite these advancements, challenges like legislative barriers and the lack of consistent political direction continue to impede progress in the telehealth domain​ [47].

The Asia-Pacific region anticipates a surge in telehealth adoption driven by digital demand and pandemic-induced behavioral changes, while South East Asia exhibits widespread telehealth growth across healthcare aspects [48]. The telehealth adoption across the Asia-Pacific region has shown remarkable growth between 2019 and 2021 and is projected to continue rising by 2024. China’s adoption nearly doubled to 47% and is expected to reach 76%. Indonesia’s usage more than doubled to 51%, with a forecast of 72%. Malaysia and the Philippines both anticipate reaching a 70% adoption rate, increasing from 30% to 29%, respectively. India’s adoption is projected to more than double to 68%, while Singapore, which had a significant increase from 5 to 45%, is expected to achieve a 60% adoption rate. This trend indicates a robust uptake of telehealth services in the region [48].

## Global telemedicine and E-pharmacy policy dynamics

In the context of telemedicine and e-pharmacy regulations within South East Asia, a notable distinction emerges with Singapore, Malaysia, and Indonesia being the only countries to have formalized legal frameworks governing both telemedicine practices and the dissemination of electronic information. In these countries, tele-consultation is restricted to patients already under the care of healthcare practitioners or as part of ongoing treatment, specifically in Singapore and Malaysia. Additionally, for scenarios requiring more intensive medical intervention, such as new referrals, emergency cases, or invasive procedures, both Malaysia and Indonesia mandate physical presence and face-to-face consultations, emphasizing a cautious and regulated approach to remote healthcare. In Malaysia, the regulations further stipulate that online prescriptions, excluding narcotics and psychotropic substances, are permissible solely under the continuation of care model, reflecting a judicious use of digital prescription services [49].

In Central and Eastern Europe (CEE), telemedicine has experienced substantial growth, primarily catalyzed by the COVID-19 pandemic, which necessitated rapid advancements in technology and alterations in healthcare practices. The region’s robust digital infrastructure, coupled with the innovative drive of local companies and the challenges posed by an aging demographic, has significantly contributed to this expansion. According to the European Commission’s Market Study on Telemedicine, the global telemedicine market was projected to grow annually by 14% by 2021, a rate that was likely surpassed due to the pandemic’s impact. More specifically, the Europe Telehealth Market, valued at US $6,185.4 million in 2019, is anticipated to witness an annual growth rate of 18.9% from 2020 to 2030. This trend underscores the increasing reliance on and potential of telemedicine in addressing healthcare needs in the CEE region [50].

In the Middle East, telehealth and telepharmacy, have seen varied degrees of adoption and progress. Despite attempts to reform healthcare delivery in the region, the progress of telemedicine has been somewhat slow, with certain expectations yet to be fully realized. However, there has been notable development in the use and adoption of these technologies [51]​. In a survey comparing the utilization of digital-health applications in Saudi Arabia and the United Arab Emirates (UAE), it was observed that a higher percentage of Saudi participants have utilized online pharmacy services (48%) compared to the UAE (36%). Conversely, awareness of teleconsultation services without prior use was higher in the UAE (43%) than in Saudi Arabia (35%). Retention data indicates that a significant proportion of users in both countries continue to engage with these services, with 80% of Saudi participants and 71% of UAE participants using teleconsultations at varying frequencies. Notably, a substantial majority of users in Saudi Arabia reported regular use of online pharmacies (90%), slightly higher than the UAE (78%), reflecting robust ongoing engagement with these digital health modalities. Notably, consumer adoption of telehealth products is primarily driven by time savings (48%) and convenience (47%), with 24-hour accessibility and efficacy both influencing 34% of users. Affordability and personal recommendations are also notable factors, while a wide range of options and quality are lesser but relevant considerations [52].

In response to the COVID-19 pandemic, a cross-sectional study was conducted among 391 licensed community pharmacists in the United Arab Emirates to assess the adoption and impact of telepharmacy services. The study revealed a predominant use of telepharmacy services, particularly via phone (95.6%) and messaging applications (80.0%). The findings highlighted that pharmacies with more pharmacists and those operating as part of a group or chain were more likely to implement a diverse range of telepharmacy services. The study identified significant barriers to telepharmacy adoption in individual pharmacies, including limited time, inadequate training, and financial constraints. There was a noticeable shift in service provision during the lockdown, with an increased reliance on telepharmacy, especially among pharmacies serving 50–100 patients per day. However, a reduction in services such as managing mild diseases and selling health products was observed during the lockdown period. The study concluded that telepharmacy played a pivotal role in supporting community pharmacies during the pandemic, with its expansion facilitated by the UAE’s advanced internet infrastructure, supportive health policies, and widespread digital connectivity [53]. Collectively, these insights reflect a global shift towards integrating and enhancing telehealth services as a response to emerging healthcare needs and technological advancements.

**Fig. 3:**
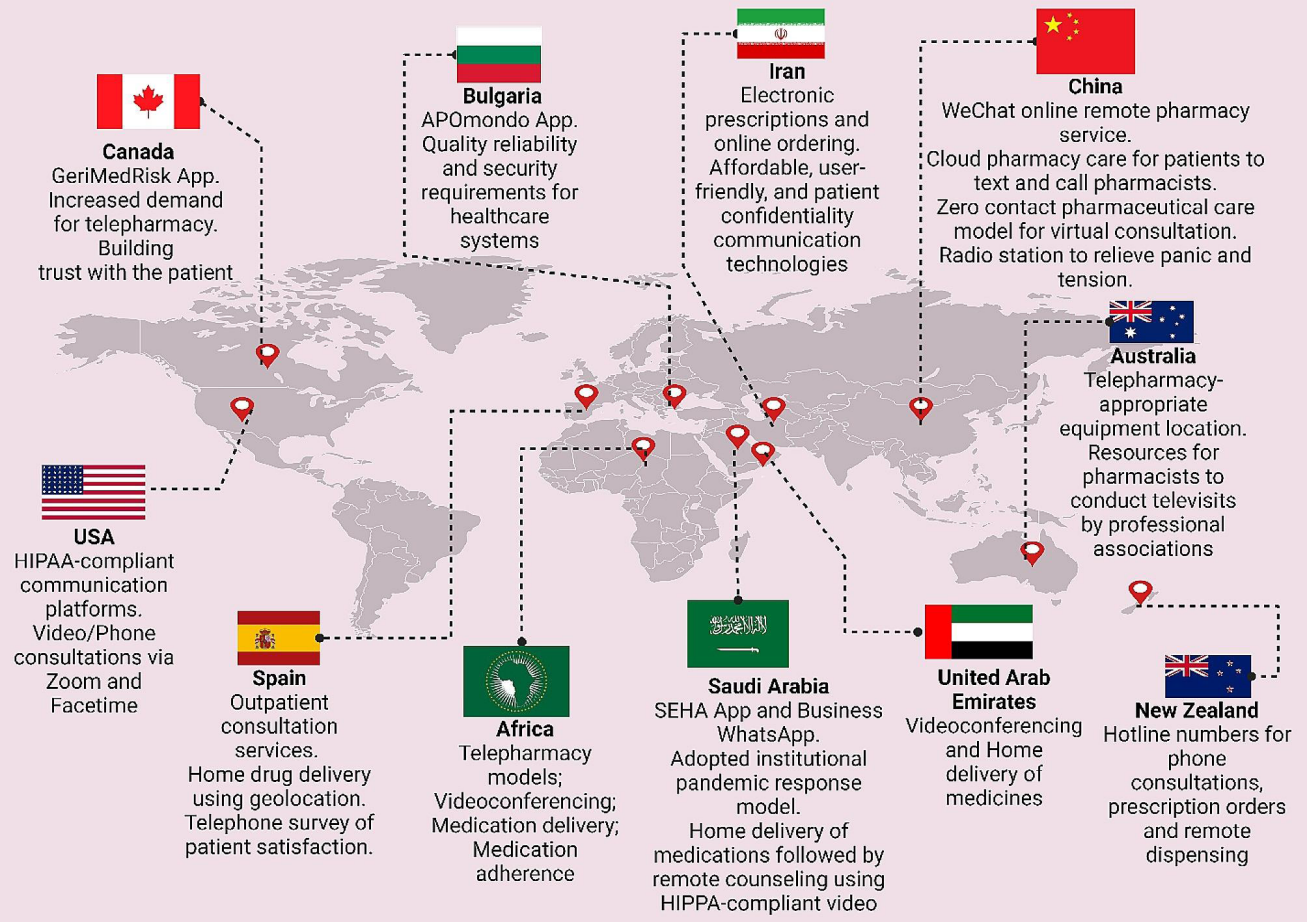
The global distribution of telepharmacy programs with an analysis of geographical distribution, technological applications, and associated benefits

Unni et al. [54] provided an extensive review of telepharmacy initiatives adopted globally during the COVID-19 pandemic. Predominantly, virtual consultations were utilized to enable at-risk patients and others to remotely access pharmacists, thereby monitoring chronic illnesses, optimizing medication usage, and providing educational support [55]. Home delivery of medicines was widely implemented to decrease the necessity for in-person visits and mitigate exposure risks [56]. Additionally, patient education was prioritized to ensure effective management of health conditions from a distance [57]. Notably, a network of hospitals in China developed cloud-pharmacy care, allowing patients to consult pharmacists via text and the internet, while Spain utilized information and communication technologies for remote pharmaceutical care [58; 59]. Zero-contact pharmaceutical care, introduced in China, facilitated online medication consultations, eliminating direct contact [60]. The Kingdom of Saudi Arabia and other regions adapted new e-tools and teleprescriptions to enhance service accessibility [61]. The U.S. focused on credentialing pharmacists for telehealth to ensure competent service provision, and New Zealand implemented hotline numbers for phone consultations to further reduce physical visits [62; 63]. These initiatives reflect a significant shift towards innovative, technology-driven solutions in pharmaceutical care during a global health crisis. Refer to Fig. 3 for a graphical depiction of the worldwide distribution and applications of telepharmacy initiatives.

## Tracing the Private Sector’s Impact on Healthcare’s Technological Transformation

### The role of the private Sector in the fourth industrial revolution

The World Economic Forum underscores the private sector’s leading role in digital inclusion and the acceleration of actions pertinent to the Fourth Industrial Revolution. This revolution affects economies, industries, and global issues profoundly, indicating the private sector’s critical role in driving technological advancements and digital platforms that deliver impactful healthcare solutions [64].

### Mapping digital transformation in healthcare

A comprehensive analysis performed by Dal Mas et al. [65] meticulously maps the intricate terrain of digital transformation in healthcare, spotlighting the private sector’s instrumental role. Initially, the investigation encompassed an extensive array of diverse studies, leading to the identification of five main areas of digital technologies: smart health technologies, data-enabled and data collection technologies, Industry 4.0 tools and technologies, cognitive technologies, and drug & disease technologies. These domains frame the future research pathways, primarily steered by the private sector’s innovative drive. A significant proportion of the literature addresses healthcare broadly, suitable for both private and public sectors, yet a notable segment specifically focuses on the private sector’s endeavors, with a pronounced emphasis on the pharmaceutical domain [66; 67].

### Public-private partnerships in healthcare delivery

The highlighted technologies, including digital platforms and telemedicine, exemplify the private sector’s trailblazing contributions to digital healthcare advancements. For instance, public-private partnerships (PPP) in India have emerged as a pivotal model for realizing universal healthcare (UHC), especially against the backdrop of acute healthcare shortages and urban-rural divides. Notably, mega PPP projects have successfully deployed technology-enabled remote healthcare (TeRHC), demonstrating its feasibility and impact in reaching isolated communities. These initiatives, overcoming various challenges, serve as a compelling example for global adoption, underscoring the transformative role of PPP in healthcare delivery [68].. Furthermore, a considerable majority of the literature in telemedicine underscores the necessity for profound research implications, yet a significant minority suggests policy implications [69; 70], reflecting a complex synergy between the private and public sectors in sculpting the digital healthcare framework [71]. This synthesis underscores the private sector’s critical influence in propelling the digital transformation in healthcare, charting a course that progressively fuses technological innovation with healthcare provision.

A study highlights Indonesia’s strategic initiatives to capitalize on telehealth business opportunities, driven by the Ministry of Research and Technology’s robust support for Technology-Based Start-up Company schemes [72]. With a demographic boon of 298 million from 2020 to 2024, escalating non-communicable diseases (71%), and a growing base of 222.4 million JKN participants, the stage is set for transformative growth. Despite a critical shortage of health workers (0.4 doctors per 1000 population), the enthusiasm for telemedicine is evident, with 71% satisfaction in hospital telemedicine and 32 million active telehealth users. The Ministry’s foresight in fostering technology start-ups, exemplified by the TEMENIN platform with its 11 health platforms, is steering Indonesia towards a future where high-quality healthcare is accessible and sustainable.

### Lab@AOR: a model for PPPs in healthcare sector

The “Lab@AOR” initiative stands as a paradigmatic example of PPPs effectuating digital transformation within the healthcare sector. This strategic collaboration, between the University Hospital of Marche and Loccioni [73], a private entity, underscores the capacity of PPPs to navigate intricate challenges, stimulate international cooperation, and contribute to the development of sustainable, patient-centric healthcare solutions. Specifically, Lab@AOR was instituted to confront the nuanced challenges associated with the robotization of healthcare service delivery, highlighting the initiative’s role in fostering technological advancement through public and private sector synergy [74]. The project illustrates the evolution of Lab@AOR through three main phases: the pioneering stage, where groundwork for collaboration was laid; the nurturing stage, where collaborative exchanges were fostered; and the harvesting stage, wherein the potential of the PPP was fully unleashed. In the pioneering stage, Lab@AOR focused on a critical healthcare service component: the in-hospital preparation of medications for oncological patients. The University Hospital of Marche identified a need for innovation to improve service quality, efficiency, and safety, while Loccioni sought a real-life setting to test and refine its robotized system, APOTECAchemo [75]. This convergence of needs led to a symbiotic partnership aiming to enhance healthcare delivery through technological advancement.

During the nurturing stage, the partnership expanded the scope of APOTECAchemo to include non-oncological medications and developed additional tools like APOTECAps for manual preparation support. This phase was characterized by intensive collaboration, knowledge sharing, and continuous innovation, demonstrating the dynamic capability of the PPP to adapt and evolve in response to emerging healthcare challenges. The harvesting stage marked the international expansion of Lab@AOR, transforming it from a local initiative to an international community focused on leveraging digitalization and robotization to improve care quality and patient-centeredness. The PPP’s growth was catalyzed by its open perspective and inclusive approach, engaging entities from various cultural and institutional contexts, and fostering a network of 31 nodes across 19 countries and 3 continents.

### Advancements in telehealth business models and frameworks

In their investigative study, Velayati et al. [76] delved into the articulation of emergent business models in telehealth and scrutinized the deployment of established frameworks across a variety of telehealth segments. The research spanned an extensive range of sectors, notably telemonitoring, telemedicine, mobile health, and telerehabilitation, alongside telehealth more broadly. The scope further extended to encompass niche areas such as assisted living technologies, sensor-based systems, and specific fields like mobile teledermoscopy, teleradiology, telecardiology, and teletreatment, presenting a thorough analysis of the telehealth landscape. Within the telemedicine and telehealth services sector, Barker et al. [77] introduced the Arizona Telemedicine Program (ATP) Model, a quintet-layer approach aimed at efficiently distributing telemedicine services throughout Arizona. Complementing this, Lee and Chang [78] proposed a four-component model specifically tailored for mobile health (mHealth) services pertaining to chronic kidney disease, focusing on offering a cost-effective platform for disease support and management. In the realm of telemonitoring, Dijkstra et al. [79] utilized the Freeband Business Blueprint Method (FBBM), which includes service, technological, organizational, and financial domains, to facilitate multiple telemonitoring services. Furthermore, the systemic and economic differences were explored in care coordination through Business to customer (B2C) and business (B2B) models for telemonitoring patients with chronic diseases, with the B2C model’s economic advantages were highlighted [80].

General telemedicine frameworks also received attention. Lin et al. [81] constructed a six-component framework analyzing major telemedicine projects in Taiwan, while Peters et al. [82] developed the CompBizMod Framework in Germany, encompassing value proposition, co-creation, communication and transfer, and value capture, designed to evaluate and enhance competitive advantages in telemedicine. In the specialized field of telecardiology, a comprehensive nine-component sustainable business model was crafted to facilitate mutual benefits for service providers and patients. This model emphasizes the importance of a holistic approach in ensuring the longevity and effectiveness of healthcare delivery within this domain [83]. Meanwhile, Mun et al. [84] presented a suite of five teleradiology business models aimed at providing effective, high-quality, and cost-efficient diagnoses.

The teletreatment sector saw innovative models from Kijl et al. [85], who designed a model for treating patients with chronic pain, focusing on the interrelation of components in the value network and the role of information technology. Complementarily, Fusco and Turchetti [86] introduced four models for telerehabilitation post-total knee replacement, emphasizing partnerships between care units and equipment suppliers to reduce costs and waiting lists. The mHealth and assisted living technology sector witnessed the introduction of a wearable biofeedback system model by Hidefjäll and Titkova [87], which employed Alexander Osterwalder’s Business Model Canvas and focused on a comprehensive commercialization process. Additionally, Oderanti and Li [88] presented a seven-component sustainable business model for assisted living technologies, aimed at encouraging older individuals to invest in eHealth services while reducing the pressure on health systems. These diverse clusters and models reflect the multifaceted nature of telehealth, each tailoring its approach to meet the unique demands of its domain. They collectively aim to optimize service delivery, stakeholder involvement, cost efficiency, and patient care quality, marking significant strides in the ongoing evolution of digital healthcare.

### Challenges and biases in healthcare technology

One key aspect is the emergence of novel medical technologies and their potential biases. These biases are often a result of insufficient consideration of patient diversity in the development and testing phases. For example, disparities in the performance of medical devices like pulse oximeters among different racial groups have been observed, potentially due to a lack of diverse representation in clinical trials. This indicates a tendency for the development of healthcare technologies that may not adequately serve all patient populations [89]. A study on the profitability and risk-return comparison across health care industries highlights the use of return on equity (ROE) as a measure of profitability from a shareholder’s perspective. This measure combines profit margin, asset utilization, and financial leverage. The study analyzed financial data of publicly traded healthcare companies, providing insights into the financial dynamics of the healthcare sector. It revealed that while companies like Pfizer Inc. and UnitedHealth Group reported similar profitability, they had substantial differences in profit margin and asset utilization, indicating diverse financial strategies within the healthcare sector. This study underscores the complexity of financial performance in healthcare, where profitability measures need to be balanced with risk assessment and the broader impact on healthcare provision​ [90].

Additionally, an article discusses the benefits, pitfalls, and potential biases in healthcare AI. It emphasizes that as the healthcare industry adopts AI, machine learning, and other modeling techniques, it is seeing benefits for both patient outcomes and cost reduction. However, the industry must be mindful of managing the risks, including biases that may arise during the implementation of AI. Lessons from other industries can provide a framework for acknowledging and managing data, machine, and human biases in AI. This perspective is crucial in understanding how the integration of advanced technologies in healthcare can be influenced by the drive for profitability and efficiency, possibly at the expense of equitable and patient-centered care [91; 92].

## Cosmeceuticals in the online pharmacy market

Cosmeceuticals, a term derived from the combination of cosmetics and pharmaceuticals, refer to a category of products that are formulated to provide both aesthetic improvements and therapeutic benefits. These products, typically applied topically, are designed to enhance the health and beauty of the skin, going beyond the mere cosmetic appearance. The exploration of cosmeceuticals in the online pharmacy market reveals a multifaceted and rapidly expanding industry. Bridging the gap between cosmetics and pharmaceuticals, they form a significant portion of the skincare industry. Cosmeceuticals are formulated from various ingredients, with their main categories being constantly discussed and analyzed in the scientific community [93]. They have taken a considerable share of the personal care industry globally, constituting a significant part of dermatologists’ prescriptions worldwide [94]. This surge is further fueled by increasing consumer demand for effective and safe products, including anti-aging skincare cosmeceuticals, a need which has been intensified by concerns over pollution, climate change, and the COVID-19 pandemic [95].

**Fig. 4:**
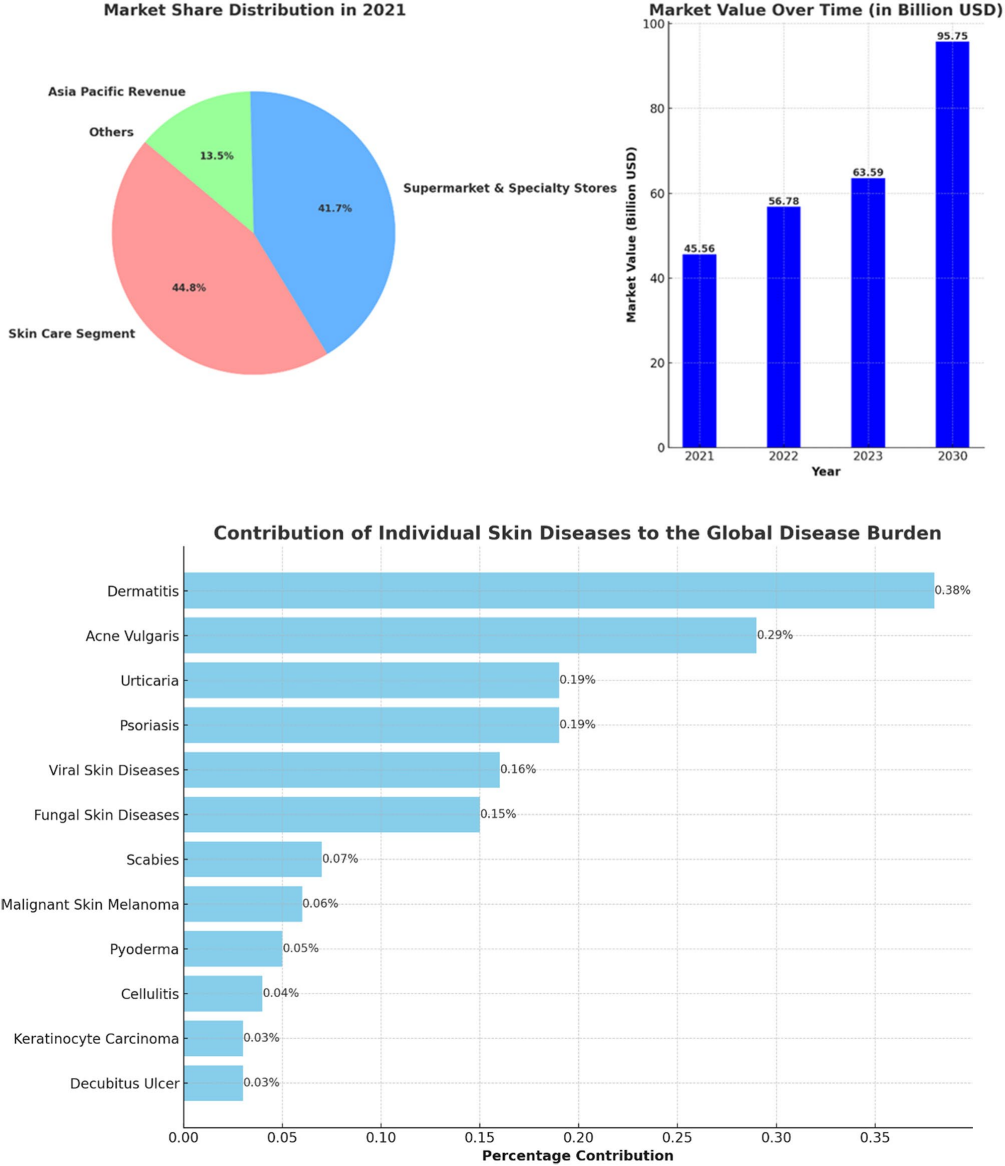
The left panel presents the market share distribution for key segments in the cosmeceuticals industry in 2021, including Skin Care Segment, and Supermarket & Specialty Stores, for Asia Pacific Revenue, with percentages for each category. The right panel displays the market value progression over time from 2021 to the projected value in 2030, with bold numbers indicating the value in billion USD for each year. The lower horizontal bar chart depicts the percentage contribution of various skin diseases to the global disease burden

The global cosmeceuticals market is experiencing robust growth. Valued at USD 56.78 billion in 2022, it’s projected to expand to USD 95.75 billion by 2030, with a compound annual growth rate (CAGR) of 7.45%. This growth trajectory is propelled by the innovative integration of bioactive ingredients known for their medical benefits​ [96]. Another report confirms this upward trend, indicating the market was worth $45.56 billion in 2021 and is on a path of significant growth to USD 114 billion by 2030. The global disease burden is significantly impacted by various skin diseases, with dermatitis, psoriasis, and acne vulgaris among the most prevalent, contributing 0.38%, 0.19%, and 0.29% respectively. The pervasive nature of these conditions drives a substantial demand for effective treatments, propelling the integration of cosmeceuticals into the online pharmacy market. This integration not only offers convenient access to a range of therapeutic skincare products but also caters to the rising consumer inclination towards self-care and preventive healthcare. As a result, the online availability of cosmeceuticals is not just addressing the immediate needs of individuals suffering from skin conditions but is also reshaping the landscape of personal healthcare by making specialized treatments more accessible and customizable [97]. See Fig. 4.

Several factors are contributing to this expansion of the cosmeceuticals market. The market is driven by innovation in natural ingredients and a significant penetration of internet, smartphone, and social media applications, which attract potential consumer populations and reflect constantly changing consumer behavior [98]​​. The cosmeceuticals market’s robust CAGR and revenue share, especially in regions like Asia Pacific, further signify its burgeoning presence and potential within the global market [99]​. Integration into online pharmacies is a key aspect of this market’s evolution, offering easier access to these products for a wider customer base. As the market continues to grow, it’s anticipated that the blend of cosmeceuticals with online pharmaceutical platforms will become increasingly seamless, offering consumers a diverse range of accessible, effective, and beneficial skincare and health products. This integration is likely to be driven by the growing trend of e-commerce and digitalization in healthcare and personal care sectors.

The landscape of online pharmacies, particularly concerning cosmeceuticals, is evolving. While the overall penetration for non-specialty drugs in mail-order and online pharmacies is low, they represent a significant portion of specialty prescription revenues at 37%. Despite this, only 13% of consumers consider these as their primary pharmacy choice, indicating a growing but still emerging market​​​​. Strategies are in place to enhance the market appeal of these pharmacies, focusing on speed, convenience, and personalized experiences, such as video telehealth visits, to attract a broader consumer base [100].

The dissertation “L’Oréal Portugal: A Digital Challenge for the Active Cosmetics Division” authored by Ascenso [101] provides an in-depth examination of the impact of digital evolution on the Portuguese cosmeceutical sector and its implications for L’Oréal, a significant cosmetics company. It posits that while L’Oréal has foundational digital competencies, the rapidly evolving digital landscape presents a broad spectrum of potential risks and opportunities. The study details the operations of L’Oréal’s Active Cosmetics Division, which manages brands predominantly sold in pharmacies and parapharmacies, and explores the potential repercussions of digitalization on L’Oréal Portugal’s strategic and operational frameworks. Furthermore, the thesis highlights the expanding role of e-pharmacies and the need for legal reforms to facilitate their operation. It discusses the prevalent trends in the cosmetic industry, such as the increasing demand for natural, male-focused, and environmentally friendly products. The dissertation scrutinizes L’Oréal’s strategic pillars, including innovation, acquisition, and regional growth, emphasizing the need for the company to integrate advanced technologies and recalibrate its business methodologies in light of digital progression [101]. Although L’Oréal has initiated some digital strategies targeting consumers and pharmacies, there’s a recognized need for an intensified focus on digital marketing aimed at clients. An exploratory attempt by L’Oréal to implement an online ordering platform for pharmacies did not meet success, indicating possible industry unreadiness for such advancements. This case study serves as a critical examination of how traditional companies in the pharmaceutical and cosmetics sectors must adapt to the digital age’s challenges and opportunities [101].

In a collaborative endeavor with L’Oréal, an associated digital agency provided a comprehensive suite of services that encompasses the full management of social media pages, the development of e-commerce websites, the establishment of Customer Relationship Management (CRM) platforms tailored for pharmacies, and the execution of digital campaigns leveraging QR codes, SMS marketing, and newsletters. These digital tools confer a competitive edge, facilitating a deeper comprehension of consumer behavior and the potential to augment value extraction from customer interactions. For the laboratories, particularly those associated with cosmetics, the advantages are twofold: an increase in sell-out figures, thereby enhancing direct sales to end consumers, and a boost in sell-in metrics, reflecting a rise in transactions to pharmacies or wholesalers. The online ordering feature, as noted by João Roma, a manager at La Roche-Posay, could result in a cacophony of processes if laboratories were to individually develop distinct methods. He advocates for the utilization of pre-existing platforms, such as the established e-learning infrastructure, to spearhead ventures into the online marketplace [101].

A survey conducted specifically for L’Oréal’s e-learning platform, cosmeticaactiva.pt [102], across the Portuguese landscape garnered responses from 324 participants, comprising 71% general pharmacists, 13% technical assistants, 8% directors, 7% individuals responsible for procurement from laboratories, and 2% beauty/cosmetic advisors. The findings from this survey underscore the pervasive adoption of digital tools within the pharmacy sector: 82% of respondents affirmed the presence of their pharmacies on social media platforms, 80% reported the use of basic management software, 64% indicated the deployment of advanced management systems, 61% were conversant with online ordering systems directed at laboratories, 38% utilized a store locator, 28% had an established website presence, and a smaller segment of 12% offered online shopping facilities.

**Fig. 5:**
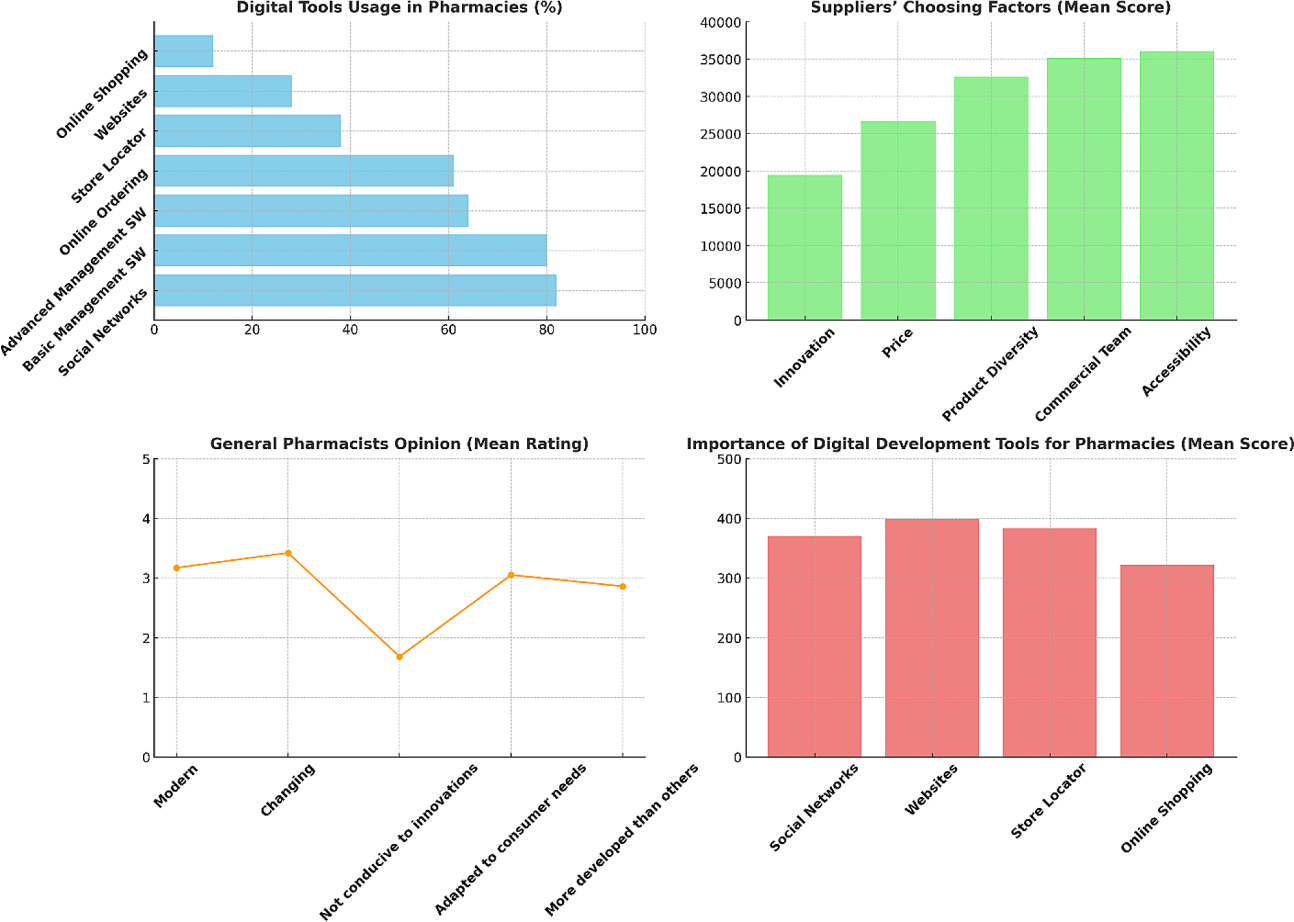
Results from Ascenso [101] survey assessing digital challenges for L’Oréal in the Portuguese cosmeceutical sector. **Digital Tools Usage in Pharmacies (upper left)**: the bar chart showing the percentage of respondents using various digital tools in pharmacies. **Suppliers’ Choosing Factors (upper right)**: the bar chart displaying the mean scores of factors that distinguish a good laboratory/supplier. **General Pharmacists Opinion (lower left)**: A line chart illustrating the mean ratings of pharmacists’ opinions on whether the pharmaceutical sector is modern, changing, conducive to innovations, adapted to consumer needs, and more developed than other sectors. **Importance of Digital Development Tools for Pharmacies (lower right)**: A vertical bar chart demonstrating the mean scores for the importance of different digital development tools for pharmacies

Another survey conducted within this study to evaluate the significance of dermocosmetic products in pharmacies yielded a mean importance rating of 4.38 out of 5, indicating that a majority of pharmacists consider these products to be highly important to their business operations. Factors critical to the differentiation of a proficient laboratory/supplier were innovation and cost-effectiveness, with mean scores of 1.9 and 2.7 respectively, on a scale from 1 (most important) to 5 (least important). A substantial majority of pharmacists, amounting to 81.8%, perceive their pharmacies as beacons of innovation and modernity. Detailed interviews elucidated that digital tools are indispensable in augmenting sales for cosmeceutical products by catalyzing demand—a dynamic not feasible with medicinal products. These tools are paramount in managing customer loyalty, facilitating enhanced communication with existing clients via online and mobile channels. Despite the challenges posed by digitalization, particularly in the realms of logistics and human resources, the management at L’Oréal is well-equipped to swiftly adapt to the evolving business landscape, as evidenced by the proactive adoption and integration of these digital strategies [101] as illustrated in Fig. 5.

The digital transformation strategies, exemplified by companies like L’Oréal, extend beyond the mere targeting of end consumers, encompassing the perspectives of various stakeholders, including retailers. This broadened focus reflects a holistic and integrated approach to digital marketing and customer engagement, indicative of a larger trend within the market. The significance of digital channels in facilitating comprehensive customer interaction and brand development is increasingly recognized. The distinction of organizations such as L’Oréal in their digital initiatives highlights the competitive advantage that can be garnered through innovative digital strategies.

The receptiveness of industry professionals, such as pharmacists, to emerging digital trends, along with the readiness of companies to engage in non-face-to-face sales models, marks a paradigm shift in traditional sales and distribution methods. This shift is reflective of a broader market trend where digital platforms are becoming integral to the customer journey. Furthermore, the potential for online sales in specialized sectors, such as dermocosmetics, and the benefits that organizations derive from the technological advancement of their client base, underscore an escalating acknowledgment of e-commerce and digital tools as crucial elements of a business strategy. This trend, with L’Oréal as a prime example, emphasizes the broader market movement towards digital transformation, not merely as an option but as a necessity for maintaining relevance and competitiveness in an ever-evolving market landscape.

## The global regulatory landscape for cosmeceuticals

Sophisticated regulatory legislation and enforcement mechanisms characterize many developed countries such as the USA, EU Member States, Canada, and Japan. These nations, along with influential organizations like the World Health Organization (WHO), significantly shape international market rules and regulations due to their market size and regulatory capacity [103]. The WHO is particularly noted for its crucial role in setting global standards, with a focus on developing and promoting international standards related to food, biological, pharmaceutical, and similar products [104]. In contrast to pharmaceuticals, the cosmetic industry necessitates a more advanced international regulatory framework due to consumers’ extensive exposure to these products. The distinction between cosmetics and pharmaceuticals varies significantly across different countries, with the USA employing a voluntary registration system for cosmetics and the EU and Japan requiring mandatory product filings prior to marketing [105]. Concerns over the safety of pharmaceutical and cosmetic products are highlighted, with an increasing consumer focus on “natural, ecological, and clean” products [106]. However, the lack of a regulatory framework for these categories underscores the need for more advanced regulations to mitigate health risks.

Intergovernmental cooperation is emphasized, with the US and EU portrayed as dominant players in the pharmaceutical and cosmetic industries, respectively. Regulatory capacity, which is essential for defining, implementing, and monitoring market rules, varies among countries and markets. This capacity depends on several factors, including staff expertise, statutory sanctioning authority, and the degree of centralization of regulatory authority [103]. The regulatory systems of the EU and US are explored, focusing on their unique approaches to medicine authorization and regulation. The European Medicines Agency (EMA) in the EU and the Food and Drug Administration (FDA) in the US serve as pivotal regulatory bodies [107; 108]. The EMA’s centralized procedure and the FDA’s premarket approval process are detailed, along with subsequent postmarket regulatory procedures. For instance, EU and US cosmetic regulations are compared, revealing differences in their approaches and the evolution of the EU’s regulatory landscape through various amendments and directives. In particular, directive 76/768/EC has been superseded by Regulation (EC) N° 1223/2009, serving as the principal regulatory framework for finished cosmetic products in the EU market. This regulation enhances product safety, optimizes the sector’s framework, and eases procedures to promote the internal cosmetic market. Incorporating recent technological advancements, including nanomaterials, it maintains an internationally acknowledged regime focused on product safety without altering existing animal testing prohibitions [109].

The Eurasian Economic Union’s (EAEU) regulatory framework for medicines and medical devices is detailed, including the legal framework established for regulating the circulation of these products. The conformity assessment methods, such as the EAC Declaration and the State Registration process, are required for manufacturers to demonstrate their products’ compliance with the standards [110]. Armenia is also part of the EAEU’s legal framework, which aims to unify regulations for the production and registration of pharmaceuticals and medical products by 2025. This unification is expected to reduce administrative costs for manufacturers and improve medicinal products for patients. Despite significant developments in the cosmetics industry, Armenia does not have an extensive regulatory framework for it. Prior to joining the EAEU, the only regulation concerning cosmetic products was the Order of the Minister of Health of the Republic of Armenia on “Hygiene Requirements of the Production and Safety of Perfume-Cosmetic Products.” Since joining the EAEU, Armenia has unified its national legislation with EAEU regulations, but there are challenges and gaps in the direct applicability of the EAEU’s technical regulations in the country [111].

In the context of the necessity for clear regulatory framework stems from two reasons. Firstly, cosmeceuticals - products straddling cosmetics and drugs - demand intensified regulatory attention. Examples include the 2007 FDA seizure of Jan Marini’s Age Intervention Eyelash, which contained the drug ingredient bimatoprost, and products boasting human stem cell cultured media, which claim rejuvenating effects but may pose safety risks due to minimal oversight [112]. A noted 1450% increase in FDA warnings (from 4 to 62 letters) between 2007 and 2011 and 2012–2017, with 8 targeting stem cell ingredient promotions, underscores the growing concern [113]. The FDA’s limited capacity to identify and assess potential drug-adulterated cosmetics raises concerns.

The second aspect focuses on the necessity for a more comprehensive and unbiased scientific and medical perspective in the FDA’s ingredient review process. The Personal Care Products Safety Act proposes a balanced committee formation including industry, consumer, and medical representatives, yet advocates for the inclusion of specialized professionals like chemists, dermatologists, toxicologists, and endocrinologists. Specific ingredients like diazolidinyl urea and quarternium-15, although effective antimicrobials, are flagged for potential skin allergy risks and formaldehyde release. The preservative 4-methylisothiazolinone, banned in Europe for rinse-off products, is noted for increasing allergic contact dermatitis cases in the US [114]. The lag in US cosmetic regulation compared to the EU is acknowledged, with the Personal Care Products Safety Act considered a significant advancement, albeit in need of further refinement [115].

The importance of consumer safety in the global regulatory landscape for cosmeceuticals, particularly for products that blur the line between cosmetics and pharmaceuticals, is a critical issue due to several key factors. Firstly, the cosmeceutical market is expanding rapidly, driven by new ingredients promising various skincare benefits like anti-aging and photoprotection. This growth necessitates clear regulatory guidelines to ensure that these products are safe and their claims are clinically proven. The FDA, for instance, differentiates between cosmetics and cosmeceuticals based on their intended use, particularly if a product is marketed as a cosmetic but functions in a way that affects the structure of the human body, classifying it as a cosmeceutical [116].

Secondly, the legal and regulatory distinctions between drugs and cosmetics are significant. Drugs are subject to FDA approval based on their intended use in treating diseases or affecting the body’s structure or function, whereas cosmetics are not. This difference becomes crucial when products are marketed with drug-like claims but are not regulated as drugs, potentially leading to consumer safety issues. For example, botanical cosmeceuticals, which contain natural ingredients like herbal extracts, need thorough evaluation to ensure consistency in therapeutic effects [117]. Additionally, cosmeceutical manufacturers must be careful with marketing and advertising claims to avoid legal implications. Misleading claims can lead to lawsuits and regulatory actions, as seen in past cases where companies faced consequences for unfounded product claims. Moreover, the FDA advises cosmeceutical manufacturers to follow Good Manufacturing Practices (GMP) to reduce the risk of misbranding or mislabeling. These guidelines include production practices and specific warning statement guidelines, emphasizing the importance of substantiating the safety of these products [118].

## The global regulatory landscape for online pharmacy

Online pharmacies pose various risks to consumers, including the potential health hazards from counterfeit or substandard medications and the inappropriate use of prescription drugs. The regulatory landscape for these pharmacies varies significantly across nations, with some countries like the United States implementing specific laws, while others, such as France, have instituted outright bans [119]. The European Union, for instance, has implemented a mandate effective from 1 July 2015, which requires member states to adhere to legal provisions for a common logo specific to online pharmacies. This is coupled with an obligation for national regulatory authorities to maintain a registry of all registered online medicine retailers, as detailed by the European Medicines Agency [120]. Furthermore, the sale of certain medications online within the EU is permissible, contingent upon the registration of the pharmacy or retailer with respective national authorities​ [121]. Additionally, the Council of Europe’s MEDICRIME Convention introduces an international treaty that criminalizes the online sale of counterfeit medicinal products, enforcing prosecution irrespective of the country in which the crime is perpetrated [122].

Switzerland presents a unique stance, where Swissmedic strongly advises against the online purchase of medicines due to the high risk of illegal sourcing and poor quality. However, Swiss mail-order pharmacies with a valid cantonal license to operate a mail-order business are exempted from this advisory​ [123]. The Swiss Mail-Order Pharmacists Association and its affiliates, such as Zur Rose AG and MediService AG, actively advocate for a modern and equitable regulation of mail-order medicine sales​ [124]. The legislative framework is further bolstered by the Federal Act on Medicinal Products and Medical Devices, which regulates therapeutic products to guarantee their quality, safety, and efficacy​ [125]. In the Middle East, community pharmacy practice is predominantly governed by national Ministries of Public Health or equivalent governmental entities, with most community pharmacies being privately owned​ [126]. The region’s involvement in the Global Cooperation Group, which encompasses various international regulatory bodies like the EMA and USFDA, signifies a collaborative approach towards drug regulatory affairs in the MENA region [127]. Despite these advances in regulatory collaboration, it is notable that currently no specific regulations have been detected for online purchases from online pharmacies in the Middle East, highlighting a significant area for potential regulatory development. Furthermore, a notable transition is observed in pharmacy education across several Middle Eastern nations, with an inclination towards introducing Pharm.D degrees to replace traditional pharmacy degrees, reflective of evolving educational standards in the pharmaceutical field [128]. This shift in education parallels the need for updated regulatory frameworks, especially in the context of the burgeoning online pharmacy sector.

Furthermore, Australia permits the sale of both Prescription-Only Medicines (POMs) and Over-the-Counter (OTC) medications online, provided that brick-and-mortar pharmacies comply with all relevant laws and practice standards [129]. In contrast, South Korea maintains a stringent stance, prohibiting the online sale of both POMs and OTC medicines, with sales confined exclusively to physical stores registered with the Regulatory Authority (RA) [130]. China, Japan, Russia, Singapore, and Malaysia exhibit a more selective regulatory framework. China and Russia allow the online sale of OTC medicines only, with China imposing additional restrictions on third-party e-commerce platforms and Russia having introduced a draft law in December 2017 to formalize this practice [131; 132]. Japan permits the online sale of certain OTC medicines, explicitly excluding specific substances such as fexofenadine and loratadine [133]. Similarly, Singapore and Malaysia endorse the online sale of specific OTC medicines only, adopting a “buyers beware” approach to caution consumers about the associated risks [134; 135]. Lastly, the legal landscapes in India and Indonesia remain ambiguous. India’s RA has effectively banned the online sale of medicinal products, yet this prohibition lacks legislative backing. Indonesia, too, grapples with unclear regulations, leaving the legal status of online pharmacies indeterminate [136].

**Fig. 6:**
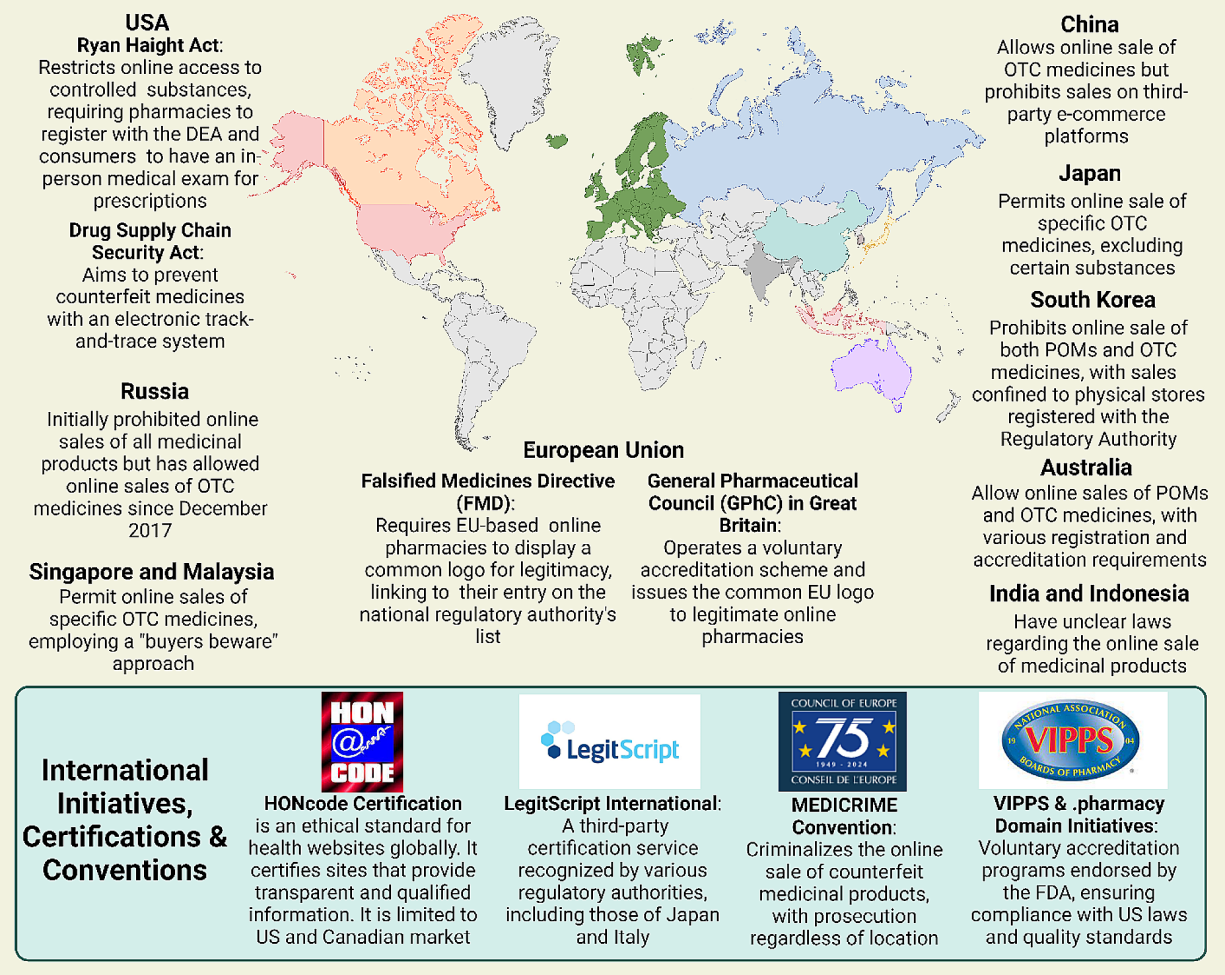
Comprehensive representation of the regulatory landscape for global online pharmacies, detailing international and national initiatives, certification programs, and conventions aimed at minimizing risks associated with the purchase of medications via online platforms

In response to these risks, several initiatives have been developed to guide and certify online pharmacies. In the United States, LegitScript offers certification to online pharmacies that comply with criteria such as appropriate licensing and registration [137]. Similarly, the Verified Internet Pharmacy Practice Sites (VIPPS) program, accredited by the National Association of Boards of Pharmacy, ensures pharmacies adhere to licensing requirements in the states where they dispense medications [138]. Internationally, the Health On the Net Foundation has introduced the HONcode, an ethical standard for health websites globally. This code certifies sites that provide transparent and qualified information. However, due to the absence of international harmonization, the HONcode’s certification is limited to US and Canadian pharmacies verified by VIPPS [139]. The lack of a harmonized international approach presents significant challenges. Consumers do not have access to a comprehensive, global repository of all certified pharmacies. The diverse certification schemes are not well articulated or interconnected, leading to consumer unawareness about their significance or existence. Moreover, enforcing standards across different legal jurisdictions is complex without a unified agreement. To enhance consumer protection, it is imperative to develop and promote a standardized, minimal international code of conduct for online pharmacies. Such a code would unify requirements and allow all initiatives to clarify their roles under a common framework. Adequate oversight in the borderless online pharmacy market can only be achieved through collaborative efforts. To visualize the infographic of the global regularity landscape for the online pharmacy see Fig. 6.

## Technological innovations and Future trends in global pharmacy

The global pharmacy sector is undergoing a transformative shift, driven by the rapid advancement of technological innovations. As the world becomes increasingly digital, the integration of cutting-edge technologies like Artificial Intelligence (AI) and blockchain is setting the stage for a new era in pharmaceutical care and management. These advancements promise to revolutionize the industry by enhancing efficiency, accuracy, and security, ultimately leading to improved patient outcomes and a more streamlined healthcare experience [140].

Walgreens, in partnership with Medline, a telehealth firm, has developed a platform for patient interaction with healthcare professionals via video chat. AI’s role extends to inventory management in retail pharmacies, allowing pharmacists to predict patient needs, stock appropriately, and use personalized software for patient reminders. Although not all inventory management software in retail pharmacies utilizes AI, some, like Blue Yonder’s software developed for Otto group, demonstrate the potential of AI in predicting product sales with high accuracy, thus enhancing supply chain efficiency [141; 142]. At the University of California San Francisco (UCSF) Medical Center, robotic technology is employed to improve patient safety in medication preparation and tracking. This technology has prepared medication doses with a notable error-free record and surpasses human capabilities in accuracy and efficiency. It prepares both oral and injectable medicines, including chemotherapy drugs, freeing pharmacists and nurses to focus on direct patient care. The automated system at UCSF receives electronic medication orders, with robotics handling the picking, packaging, and dispensing of individual doses. This system also assembles medications on bar-coded rings for 12-hour patient intervals and prepares sterile preparations for chemotherapy and intravascular syringes [143].

In the realm of global pharmacy, blockchain technology emerges as a pivotal force, driving advancements across various facets of healthcare and pharmaceuticals. At the forefront of its application is the enhancement of supply chain transparency [144]. Blockchain’s immutable ledger ensures the provenance and legitimacy of medical commodities, offering an unprecedented level of visibility from manufacturing to distribution. This is particularly vital in areas plagued by counterfeit drugs, where systems like MediLedger are instrumental in verifying the legality and essential details of medicines [145].

The utility of blockchain extends to the implementation of smart contracts — scripts processed on the blockchain that bolster transparency in medical studies and secure patient data management [146]. These contracts find extensive use in advanced medical settings, as evidenced by a blockchain-based telemonitoring system for remote patients and Dermonet, an online platform for dermatological consultation [147].

Furthermore, blockchain is revolutionizing patient care through patient-centric Electronic Health Records (EHRs). By decentralizing EHR maintenance, blockchain empowers patients with secure access to their historical and current health records [148]. Prototypes like MedRec and systems such as MeD Share exemplify how blockchain can provide complete, permanent access to clinical documents and facilitate the sharing of medical data between untrusted parties, respectively, ensuring high information authenticity and minimal privacy risks [149; 150]. In verifying medical staff credentials, blockchain again proves invaluable. Systems like ProCredEx, based on the R3 Corda blockchain protocol, streamline the credentialing process, offering rapid verification while allowing healthcare entities to leverage their existing data for enhanced transparency and assurance about medical staff experience [151].

The integration of blockchain with Internet of Things devices for remote monitoring marks another leap forward, significantly bolstering data security. By safeguarding the integrity and privacy of patient data collected by these devices, blockchain mitigates the risk of tampering and ensures that only authorized parties can access sensitive information [152]. Besides, a blockchain-based drug supply chain initiative, PharmaChain, utilizes AI for approaches against drug counterfeit and ensures the drug supply chain is more traceable, visible, and secure. For online pharmacies, this means a more reliable supply chain and assurance of drug authenticity, crucial for maintaining trust and safety [153].

In response to the COVID-19 pandemic, the PharmaGo platform has emerged as an innovative solution in Sri Lanka, revolutionizing the delivery of pharmacy services. As traditional pharmacies grapple with the challenges of meeting all customer needs in one location, PharmaGo addresses this by providing a comprehensive online pharmaceutical service. It allows customers to access a wide range of medications through a single platform, reducing the need to visit multiple pharmacies. Utilizing image processing technology, pharmacy owners can accurately identify prescribed medicines, while the system’s predictive analytics forecasts future drug demands, enhancing stock management. Additionally, PharmaGo’s AI-powered medical chatbot offers real-time guidance, ensuring a seamless and efficient customer experience. This platform represents a significant advancement in healthcare accessibility and pharmacy service delivery in the pandemic era [154]. In the same context, ontology-based medicine information system, enhancing search relevance through a chatbot interface was presented by Amalia et al. [155]. Addressing conventional search engines’ limitations in interpreting data relationships, it employs semantic technology to represent metadata informatively. The ontology as a knowledge base effectively delineates disease-medicine relationships, with evaluations indicating a 90% response validity from the chatbot, offering a robust reference for medical information retrieval and its semantic associations.

## Future trends for the digital transformation of in the pharmaceutical sector

Future trends for the digital transformation of pharmacies globally are heavily influenced by the transformative impact of digital technologies on healthcare delivery. The integration of telemedicine, electronic health records, and mobile health applications is pivotal in enhancing patient care. These technologies are instrumental in improving data sharing and collaboration among healthcare professionals, increasing the efficiency of healthcare services. Additionally, they offer significant potential for personalized medicine through data analytics and play a crucial role in patient engagement and self-management of health. The importance of these technologies in creating a more connected and efficient healthcare system is underscored, marking a significant shift in the global healthcare landscape [156].

In the pharmaceutical sector, the COVID-19 pandemic has catalyzed a significant shift towards Pharmaceutical Digital Marketing (PDM), particularly for over-the-counter drugs. This shift focuses on utilizing online pharmacies and digital platforms for targeted advertising, directly reaching consumers. The trend towards purchasing OTC drugs online has grown, driven by the convenience and efficiency of digital channels. While PDM faces challenges like regulatory constraints and the need for digital proficiency, it offers substantial opportunities in enhancing customer engagement and precise marketing. The future of PDM is poised to be more consumer-centric, integrating advanced technologies like AI, and emphasizing personalized marketing strategies to strengthen brand engagement and customer interaction [157].

Artificial intelligence holds immense potential to revolutionize the field of pharmacy, offering numerous benefits that can significantly enhance efficiency and patient care. One of the primary applications of AI in this sector is the automation of routine tasks. By utilizing AI, pharmacies can automate critical processes such as prescription processing, checking for drug interactions, and managing inventory. This automation not only streamlines operations but also minimizes the likelihood of human error, thereby increasing the overall efficiency of pharmacies [158].

Furthermore, AI can play a pivotal role in personalized medication management. This is particularly beneficial for patients with chronic conditions such as diabetes who require careful management of their insulin dosages, as fluctuations in blood sugar levels can lead to serious complications. AI systems can monitor patients continuously, provide timely reminders for medication intake, and dynamically adjust treatment plans based on individual health data. Such personalized management ensures that patients receive optimal care tailored to their specific needs, potentially improving treatment outcomes. Incorporation of AI into electronic health records presents another significant advancement. By integrating AI with EHRs, healthcare providers can access real-time patient data. This integration empowers healthcare professionals to make more informed care decisions, enhancing the quality of patient care. Moreover, it significantly reduces the likelihood of medication errors, a critical concern in healthcare.

Likewise, AI’s capability to analyze extensive patient data is invaluable. It can identify patterns and trends in medication adherence, detect potential drug interactions, and pinpoint adverse drug reactions. These insights are crucial for healthcare professionals and researchers. By understanding these patterns, they can develop more effective medication adherence strategies and support systems, contributing to better patient outcomes and advancing the overall field of pharmaceutical care.

In the expansive realm of chemical space, the pharmaceutical industry faces the continual challenge of identifying new active pharmaceutical ingredients (APIs) for diverse diseases [159]. High throughput screening (HTS), despite its advancements in recent decades, remains resource-intensive and often yields unsuitable hits for drug development. The failure rate of investigational compounds remains high, with a study citing only a 6.2% success rate for orphan drugs progressing from phase I to market approval [160, 161].

Machine learning presents a transformative approach to this challenge. It offers an alternative to manual HTS through in silico methodologies. ML-driven drug discovery boasts several advantages: it operates continuously, surpasses the capacity of manual methods, reduces costs by decreasing the number of physical compounds tested, and early identifies negative characteristics of compounds, such as off-target effects and sex-dependent variability [162].

A substantial advancement in the realm of machine learning has emerged from major pharmaceutical entities, notably AstraZeneca, in conjunction with research institutions. This progress is evidenced by the development of an innovative algorithm that demonstrates both time efficiency and effectiveness in the sphere of drug discovery. The recent introduction of this algorithm significantly enhances the process of determining binding affinities between investigational compounds and therapeutic targets. It surpasses traditional in silico methods in terms of performance. The application of this algorithm underscores the remarkable potential of machine learning in accelerating the identification and development of novel therapeutic agents [163].

Moreover, the proficiency of machine learning in managing vast and intricate datasets has rendered it indispensable in research focused on cancer targets, utilizing diverse and extensive datasets. This approach is fundamental in numerous drug discovery initiatives, especially those targeting various forms of cancer. A wide array of ML techniques, ranging from supervised to unsupervised learning, are employed to discern chemical attributes that are indicative of potential therapeutic efficacy against a spectrum of cancer targets. This methodology is crucial in identifying novel compounds that could be effective in cancer treatment, leveraging the rich and complex data available in oncological research [164].

## Conclusion

The digital transformation in the pharmacy sector is significantly reshaping healthcare delivery, driven by the integration of cutting-edge technologies like Artificial Intelligence and blockchain. This transformation is marked by a substantial growth in the digital pharmacy market, with a projected annual growth rate of 14.42%, leading to a market volume of approximately $35.33 billion by 2026​​.

One major aspect of this transformation is the growing reliance on online pharmacy platforms, largely influenced by the COVID-19 pandemic. Consumer trust in online medication purchases has significantly increased, indicating a shift towards digital healthcare solutions. The adoption of telehealth services, including telepharmacy, has surged, with patient adoption in the United States increasing from 11% in 2019 to 46%. This shift towards digital-first services enhances convenience and access to care but also introduces regulatory challenges, particularly in maintaining patient safety and quality standards in the rapidly evolving online healthcare environment​​.

The cosmeceuticals market, a segment within online pharmacies, is experiencing robust growth. Cosmeceuticals, which bridge the gap between cosmetics and pharmaceuticals, have become a significant part of the skincare industry. The market, valued at USD 56.78 billion in 2022, is projected to expand to USD 95.75 billion by 2030. This expansion is driven by factors like innovation in natural ingredients and significant penetration of internet, smartphone, and social media applications. Despite the growth, the overall penetration for non-specialty drugs in mail-order and online pharmacies remains low, representing a significant portion of specialty prescription revenues. The evolving landscape of online pharmacies in the cosmeceuticals sector reflects a trend towards more accessible and customizable personal healthcare solutions​​.

Technological innovations are setting the stage for a new era in pharmaceutical care and management. AI’s role extends to areas like inventory management in retail pharmacies, where it predicts patient needs and enhances supply chain efficiency. Blockchain technology enhances supply chain transparency and legitimizes medical commodities, especially crucial in areas affected by counterfeit drugs. Blockchain also plays a vital role in patient-centric Electronic Health Records and telemonitoring systems. For instance, PharmaGo, an innovative platform developed in response to the pandemic, provides a comprehensive online pharmaceutical service, demonstrating the significant advancements in healthcare accessibility and pharmacy service delivery​​.

These technological advancements are instrumental in improving data sharing and collaboration among healthcare professionals. They offer significant potential for personalized medicine through data analytics, playing a crucial role in patient engagement and self-management of health. The future trends in the pharmaceutical sector, particularly influenced by the COVID-19 pandemic, indicate a shift towards Pharmaceutical Digital Marketing (PDM) and a more consumer-centric approach. AI’s potential in revolutionizing pharmacy includes automation of routine tasks, personalized medication management, real-time patient data access, and the identification of patterns in medication adherence and potential drug interactions​​.

## Data Availability

No datasets were generated or analysed during the current study.
